# Posterior Reversible Encephalopathy Syndrome (PRES) and Drug-Induced Hypersensitivity Syndrome (DIHS) following Immunotherapy and BRAF/MEK Inhibition with Continued Response in Metastatic Melanoma

**DOI:** 10.1155/2021/8845063

**Published:** 2021-05-12

**Authors:** J. M. Sabile, D. J. Grider, K. A. Prickett, H. Li, P. V. Mallidi

**Affiliations:** ^1^Virginia Tech Carilion School of Medicine, 2 Riverside Circle, Roanoke, VA 24016, USA; ^2^Dominion Pathology Associates, 1 Riverside Circle, Roanoke, VA 24016, USA; ^3^Carilion Clinic-Dermatology, 1 Riverside Circle, Suite 300M, Roanoke, VA 24016, USA; ^4^Carilion Clinic-Radiology, 1906 Belleview Ave SE, Roanoke, VA 24014, USA; ^5^Blue Ridge Cancer Care, 2013 S Jefferson St, Roanoke, VA 24014, USA

## Abstract

*Background*. The role of immunotherapy continues to evolve across both solid and hematologic malignancies. However, while use of immunotherapy has increased via the advent of checkpoint inhibition, chimeric antigen receptors, and vaccines against malignant cells, there remains uncertainty regarding the recognition and management of delayed immune-related reactions and post treatment immune-related sensitivity to subsequent medications, such as BRAF/MEK kinase inhibitors. Furthermore, it is unclear how immunotherapy may alter the adverse effect profile and efficacy of subsequent lines of treatment. *Case Presentation*. Discussed is a patient with stage IV metastatic melanoma who failed first-line treatment with a combination of nivolumab and ipilimumab. He was then treated with BRAF/MEK kinase inhibition via Encorafenib and Binimetinib. Shortly thereafter, the patient developed posterior reversible encephalopathy syndrome (PRES) and a generalized pruritic rash that was biopsied with consideration toward drug reaction versus drug-induced hypersensitivity syndrome (DIHS), formerly called drug reaction with eosinophilia and systemic symptoms (DRESS). The BRAF/MEK combination was held and steroid taper initiated with continued response even beyond conclusion of the steroid taper. *Discussion and Conclusions.* This case highlights the diagnostic challenge presented by PRES and DIHS in the setting of immunotherapy and BRAF/MEK kinase inhibition for malignant melanoma. The clinical rationale for reinitiating therapy following severe immune reactions subsequent to immunotherapy in the setting of relapsed/refractory metastatic melanoma is discussed. Additionally, the durable response our patient experienced throughout the drug hold period and steroid taper and its clinical potential etiologies and applications are reviewed. As checkpoint inhibition and tyrosine-kinase inhibitors have become cornerstones of cancer therapy, larger studies and long-term observations are needed to investigate the risks and benefits across different sequences of therapy.

## 1. Introduction

Over the last twenty years, immunotherapy has asserted its role as a critical constituent in the treatment of advanced and relapsed/refractory malignancy. Specifically, the advent of both checkpoint inhibitors anti-CTLA4 (ipilimumab) and anti-PD1 (pembrolizumab and nivolumab) has resulted in nearly half of all stage IV metastatic melanoma patients achieving cure [1]. In parallel, the use of BRAF and MEK inhibitors to selectively target the action of BRAF V600 E and K mutations, found in approximately half of malignant melanomas, has become standard of care. The most efficacious sequence of these modalities has been subject to debate [2, 3].

However, while immunotherapy has yielded improved outcomes across various solid tumors and hematologic malignancies, there has also been an increase in adverse events related to immunotherapy. Immune-related adverse events may occur soon after starting treatment but may also be delayed. These events may occur days, months, or years later often posing a diagnostic conundrum for clinicians [4].

Furthermore, while many delayed immune-related adverse events are not life-threatening, such as vitiligo and alopecia, others represent actionable conditions requiring intervention, such as adrenal insufficiency, myocarditis, pericarditis, colitis, and Guillain-Barré Syndrome. Additionally, there have been recent reports of rarer, life-threatening adverse events occurring postimmunotherapy such as drug-induced hypersensitivity syndrome (DIHS) and posterior reversible encephalopathy syndrome (PRES) [4–6]. To complicate this clinical trend, there have also been reports of continued clinical benefit, long after the discontinuation of both immunotherapy and MEK/BRAF therapy [7].

## 2. Case Presentation

**Figure 1:**
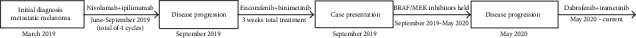
Visual timeline of treatment course leading up to symptoms present during case presentation.

**Figure 2:**
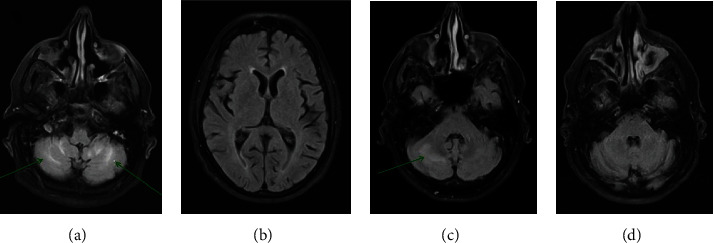
T2-FLAIR Magnetic Resonance Imaging (MRI) brain with contrast demonstrating (a) symmetric T2-hyperintensity of the cerebellum on initial case presentation, (b) representative image demonstrating lack of cerebral involvement during initial case presentation, (c) subsequent 2-week follow-up MRI demonstrating resolving T2-hyperintensity in the cerebellum, and (d) complete resolution of cerebellar T2-hyperintensity 6 months postpresentation.

**Figure 3:**
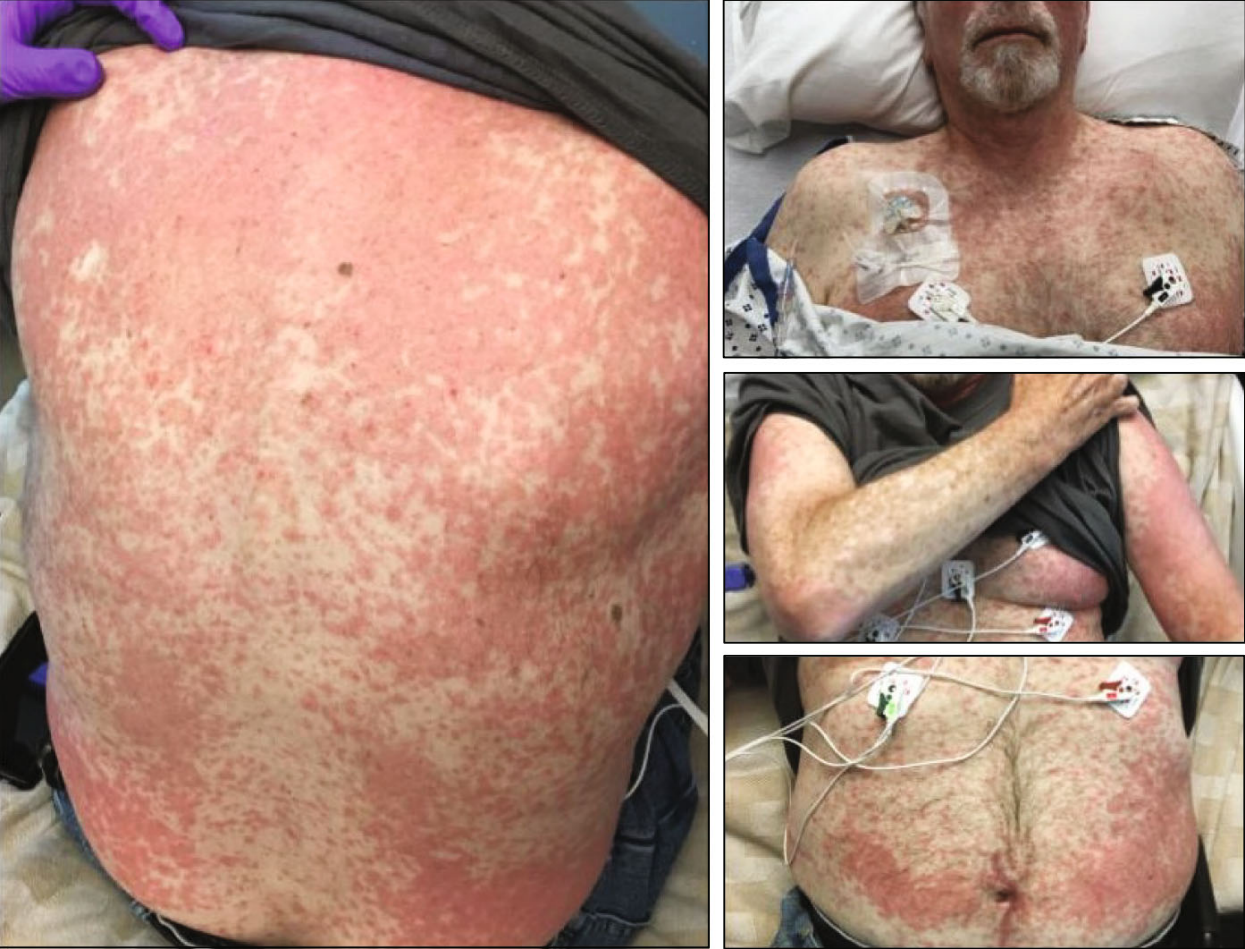
Generalized pruritic and confluent morbilliform rash involving the face, neck, chest, back, abdomen, arms, thighs, and lower legs. Erythema and mild edema on the cheek, ears, and neck.

**Figure 4:**
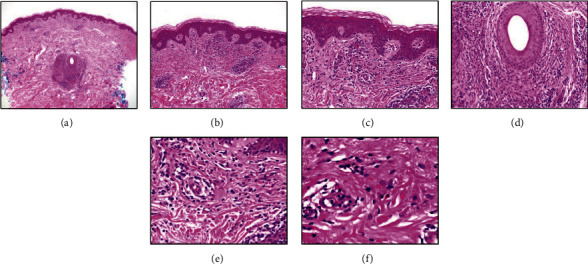
(a) A low-power view showing mild upper dermal perivascular and perifollicular inflammation (H&E 40 magnification), (b) basketweave cornified layer and a upper dermal perivascular and interstitial inflammatory infiltrate (H&E 100 magnification), (c) closer view of the perivascular and interstitial inflammatory infiltrate without significant interface or epidermal abnormality (H&E 200 magnification), (d) closer view of the perifollicular inflammation (H&E 200 magnification), (e) closer view of the dermal inflammatory infiltrate of mostly small lymphocytes and a few eosinophils, consistent with drug reaction (H&E 400 magnification), and (f) high-power view of the dermal inflammatory infiltrate of lymphocytes and a few eosinophils (H&E 600 magnification).

A 68-year-old Caucasian male presented in May 2019 with small bowel obstruction requiring small bowel resection with pathology demonstrating malignant melanoma small bowel positive for BRAF V600E mutation and negative for C-Kit, PDGFRA, and KRAS mutation. Further work up showed no brain metastasis on MRI and incidental bilateral mastoiditis. Whole-body PET/CT showed multiple areas of FDG uptake including R axillary lymph node, left anterior chest wall mass, right lower lobe lung lesion, and mesenteric uptake. LDH and comprehensive metabolic panel are normal. Combination immunotherapy with nivolumab and ipilimumab was initiated in June 2019, with rapid progression during the fourth cycle in September. He was subsequently transitioned to Encorafenib and Binimetinib that same month (Figure 1). He tolerated the combination well until developing headaches, eye pain, blurry vision, and ataxia 3 weeks later. There was no history of seizures. MRI of the brain demonstrated a new, grossly symmetric, abnormal T2 hyperintense signal in the cerebellum bilaterally without significant mass effect or enhancement and without cerebral involvement (Figure 2). Lumbar puncture and CSF analysis was performed which was positive for elevated protein, but negative for white cells and culture growth. Four days from the onset of neurologic symptoms, the patient also began experiencing fevers, chills, and a diffuse pruritic rash involving the back, chest, abdomen, and bilateral proximal arms (Figure 3). Notable lab abnormalities coinciding with the onset of the rash included elevated creatinine (3.24 mg/dL), decreased GFR (18 mL/min/1.73 m^2^), and elevated liver enzymes (alkaline phosphatase 257 U/L, AST 235 U/L, and ALT 103 U/L). Serum eosinophils were normal. Anti-nuclear antibody (ANA) was negative. Hepatitis panel demonstrated positive hepatitis A IgM antibody but with negative hepatitis B and C screens. The patient was clinically asymptomatic despite positive hepatitis A IgM with unclear timing of exposure. A punch biopsy from rash on the abdomen (Figure 4) demonstrated basketweave cornified layers of skin, supporting an acute rash, with vacuolar alteration at the dermal-epidermal junction and moderate perifolliculitis. Additionally, there was a mid and upper dermal perivascular lymphohistiocytic infiltrate with a few eosinophils and neutrophils and eosinophils in the interstitium. DIHS was considered in the differential diagnosis and could not be ruled out given RegiSCAR grading of 3–4 [8]. Overall, the patient was assessed to have experienced PRES based on his neurologic symptoms and MRI results as well as a severe DIHS based on the clinical presentation of rash and dermal biopsy results. The rash was not found to be consistent with Stevens-Johnson Syndrome/Toxic Epidermal Necrolysis (SJS/TEN) given the lack of painful skin, mucosal involvement, characteristic bullae/superficial necrosis, and discordant histopathologic features [9].

High-dose prednisone was initiated at 1 mg/kg per day with intention for close monitoring and slow taper. MEK and BRAF kinase inhibitors were held on admission. Liver function and creatinine normalized through hospitalization, and rash resolved with steroid treatment. Hepatitis A was not treated given he remained clinically asymptomatic and with resolving transaminitis.

Despite not being on active therapy, the tumor continued to decrease in size clinically and radiographically. As he remained asymptomatic with no clear evidence of disease, he chose to proceed with close surveillance. Following the conclusion of the steroid taper over a six-week period, there was no recurrence of rash, lab abnormalities, or PRES symptoms. Repeat disease evaluation in March 2020 showed continued tumor response without any clinical symptoms. The patient subsequently experienced relapse/progression indicated by increased size of prior left chest metastases in May of 2020 resulting in reinitiation of a different BRAF/MEK combination therapy. Because of his previous reaction, he was cautiously started on dabrafenib followed by trametinib three weeks later. To date, the patient continues to tolerate this regimen with no recurrence of transaminitis, renal dysfunction, rash, and neurologic symptoms.

## 3. Discussion

There is significant overlap with the common adverse events associated with BRAF/MEK kinase inhibitors and immunotherapy [10]. In the context of this case, it is notably challenging to attribute the patient's diffuse, pruritic rash to either the previous treatment with nivolumab and ipilimumab or treatment with Encorafenib and Binimetinib. Furthermore, the patient's renal failure/acute kidney injury is difficult to attribute since AKI is a known sequelae of DIHS and has also been demonstrated following both immunotherapy and BRAF inhibitors [11–13]. As immune-related reactions can be delayed, they may occur while the patient is on subsequent lines of therapy which can pose both a diagnostic and therapeutic dilemma. This is especially important given the current data demonstrating prolonged overall survival with the use of frontline immunotherapy prior to BRAF/MEK inhibitors in some patient populations [2].

To date, PRES has been reported as an adverse effect related to nivolumab. In the single case report outlining this experience, PRES onset was documented at 16 days post treatment of nivolumab and was clinically characterized by sudden loss of vision, headache, nausea with vomiting, and bilateral lower extremity weakness [6]. PRES onset for the patient presented in this report occurred 3-4 weeks following the patient's last dose of nivolumab and was clinically similar to those noted in the prior case report (headache, acute vision changes, and motor dysfunction). In this case, the clinical symptoms started 7-10 days following initiation of BRAF/MEK inhibitors. Because of the timing of the PRES presentation, it was difficult to attribute cause specifically to either BRAF/MEK inhibitors versus immunotherapy. There have not been major reports to suggest that PRES is an adverse effect associated with BRAF/MEK kinase inhibitors in isolation. Importantly, there is also a lack of studies outlining how prior immunotherapy may alter the expected adverse effects of subsequent medications, including BRAF/MEK kinase inhibitors in this context. While formal diagnostic criteria for PRES are elusive, the presented case report outlines clinical and radiologic findings that support the diagnosis of PRES, namely, acute onset of neurologic symptoms, focal vasogenic edema on imaging, and reversibility of clinical and/or radiologic findings [14].

Furthermore, while cutaneous drug reactions are common in the setting of both immunotherapy and BRAF/MEK kinase inhibitors individually, this case highlights a unique temporal relationship between the onset of rash and the onset of PRES symptoms. In this scenario, it is likely that the patient experienced PRES with DIHS attributed to either delayed immune reaction from immunotherapy alone or exacerbated by initiation of BRAF/MEK inhibitors soon after immunotherapy. Symptoms did not relapse following reinitiation of a different BRAF/MEK inhibitor therapy; therefore, the patient's presentation could not be attributed to BRAF/MEK inhibitor therapy alone. Importantly, it is unclear how the patient's hepatitis A status influenced this specific presentation of PRES/DIHS. Curiously, the patient remains IgM positive for hepatitis A to date and remains clinically asymptomatic. Undoubtedly, further studies and observations are required to better understand how modulated immune response may affect the adverse effect profile of other drugs in addition to their timing.

Finally, this case brings special attention to treatment reinitiation following adverse events with unclear attribution in the setting of immunotherapy and kinase inhibition. Based on prior literature, cutaneous involvement due to either BRAF/MEK inhibitor or immunotherapy can often be managed conservatively or with concomitant steroid taper prior to reinitiation [4, 5, 10, 15]. However, in the presence of life-threatening neurologic and dermatologic sequelae, such as PRES and/or DIHS, there is little guidance on how to proceed with treatment resumption or with subsequent lines of therapy. Caution is warranted in the event of DIHS, since reinitiation of the offending medication can lead to organ failure and mortality. Convoluting this dilemma are recent observations that immune-related adverse events may correlate with melanoma treatment response and efficacy [16]. Specifically, there have been reports of patients with advanced-stage melanoma with durable treatment response long after treatment discontinuation in the setting of immunotherapy [17]. While the traditional mode of thought has assumed the potential negative impact on outcomes from treatment delays, potential risk of progression should be thoughtfully balanced with the potential for delayed immune-related adverse effects on subsequent lines of therapy. This case highlights the importance for further studies to improve the understanding of the efficacy and adverse effect profile of sequential and combined therapeutic modalities involving immunotherapy.
